# Child and adolescent mental health care in Dutch general practice: time trend analyses

**DOI:** 10.1186/1471-2296-12-133

**Published:** 2011-12-01

**Authors:** Marieke Zwaanswijk, Christel E van Dijk, Robert A Verheij

**Affiliations:** 1 NIVEL, Netherlands Institute for Health Services Research, P.O. Box 1568, 3500 BN Utrecht, the Netherlands

## Abstract

**Background:**

Because most children and adolescents visit their general practitioner (GP) regularly, general practice is a useful setting in which child and adolescent mental health problems can be identified, treated or referred to specialised care. Measures to strengthen Dutch primary mental health care have stimulated cooperation between primary and secondary mental health care and have led to an increase in the provision of social workers and primary care psychologists. These measures may have affected GPs' roles in child and adolescent mental health care. This study aims to investigate the identification and treatment of child and adolescent mental health problems in general practice over a five-year period (2004-2008).

**Methods:**

Data of patients aged 0-18 years (N ranging from 37716 to 73432) were derived from electronic medical records of 42-82 Dutch general practices. Time trends in the prevalence of recorded mental health problems, prescriptions for psychotropic medication, and referrals to primary and secondary mental health care were analysed.

**Results:**

In 2008, 6.6% of children and 7.5% of adolescents were recorded as having mental health problems; 15.2% of these children and 29.4% of these adolescents were prescribed psychotropic medication; 18.9% of these children and 22.9% of these adolescents were referred, mainly to secondary mental health care. Between 2004 and 2008, the percentages of children (chi-square: 22.06; p < 0.001) and adolescents (chi-square: 9.15; p = 0.003) who were diagnosed with mental health problems increased. An increase was also found in the percentage of children who were prescribed psychostimulants (chi-square: 8.29; p = 0.004). Prescriptions for antidepressants decreased over time in both age groups (children: chi-square: 6.80; p = 0.009; adolescents: chi-square: 13.52; p < 0.001). The percentages of children who were referred to primary (chi-square: 6.98; p = 0.008) and secondary mental health care (chi-square: 5.76; p = 0.02) increased over the years, whereas no significant increase was found for adolescents.

**Conclusions:**

Although GPs' identification of mental health problems and referrals to primary mental health care have increased, most referrals are still made to secondary care. To further strengthen primary mental health care, effective short-term interventions for child and adolescent mental health problems that can be applied in general practice need to be developed.

## Background

Mental health problems are widespread among children and adolescents [1], with prevalence rates ranging from 14-22% [2-7]. These problems can negatively affect children's current functioning and may lead to mental health problems later in life [8-13]. Early identification and adequate treatment are important to prevent a negative spiral of problems, which may be more difficult to tackle in adulthood. Still, only a minority of children and adolescents with mental health problems receive specialised care [7,14-19].

In the Netherlands, general practitioners (GPs) play an important role in the care for children and adolescents. General practice is the formal point of entry into the health care system and GPs function as gatekeepers. Almost all inhabitants are registered in general practices, which are close to the community. Because care provided by GPs is covered by the basic health insurance, which is obligatory for everyone, there are no major financial constraints to the accessibility of GP care. GPs are therefore usually the first health care providers to be consulted when parents have concerns about their child's physical or mental health. The majority of children and adolescents, including those with mental health problems, visit their GP at least once a year [18,20-22].

Strategically, general practice therefore constitutes a useful setting in which child mental health problems can be identified, treated or referred to specialised care when needed. Similar conclusions have been drawn in the UK, in which GPs are also designated as gatekeepers to mental health care [23,24]. Even in countries with different health care systems (i.e. without GPs who function as gatekeepers), the majority of children and adolescents visit primary care services at least once a year [25], which underlines the importance of the primary care setting in the identification and treatment of youths with mental health problems. It is unclear however, to what extent these opportunities are being used and whether this has changed over the years.

Previous research has indicated that a substantial number of children and adolescents with mental health problems are not identified by their GP or primary care provider [18,21,24,26]. Although not all of these youths will need additional care, the limited detection of mental health problems may prevent or delay the receipt of appropriate care for the ones who do. Literature reviews have confirmed GPs' limited detection as one of the barriers on the pathway to care for children and adolescents with mental health problems [27,28].

According to the principle of stepped care [29], children and adolescents with mental health problems should preferably be treated in primary care, and should only be referred to secondary care when treatment in primary care does not have satisfactory results. Direct referral to secondary care may only be appropriate for youths with severe mental disorders or complex treatment needs. Treatment of mental health problems in general practice may constitute some kind of psychological management (e.g. counselling) or a prescription for psychotropic medication.

Several studies have shown increases in the use of psychotropic medication by youths during the last two decades [30-32], which can mainly be attributed to an increased use of psychostimulants [33]. However, since these previous data were based on pharmacy dispensing records, which include prescriptions from GPs and psychiatrists, they may not adequately reflect GPs' role in prescribing psychotropic medication.

As in other countries, such as the UK [23] and Australia [34], various initiatives to enhance the role of primary care providers in caring for people with mental health problems have been introduced in the Netherlands. These initiatives may have changed the role of GPs in child and adolescent mental health care. Dutch GPs were offered the opportunity to consult secondary mental health care professionals, and cooperation between primary and secondary mental health care was stimulated. This may have increased GPs' identification of child and adolescent mental health problems. The accessibility of primary mental health care improved, because of an increase in the supply of social workers and primary care psychologists [35] and because care provided by primary care psychologists was included in the basic health insurance package. This improved accessibility may have increased referrals to primary mental health care providers.

Another development which may have changed the role of GPs in child and adolescent mental health care was the implementation of Youth Welfare Work Offices. Since 2005, these offices were supposed to function as the only entrance point into child mental health care. GPs were no longer expected to refer children and adolescents to mental health care directly, except when they suspected the patient to have a serious psychiatric disorder [36]. Data about the percentages of Dutch children and adolescents being referred to primary and secondary mental health care are lacking, however.

To investigate the extent to which child and adolescent mental health problems are identified and treated in general practice and whether this has changed over the years, this paper presents a comprehensive overview of mental health care as delivered by Dutch GPs between 2004 and 2008. We will investigate the rate at which different mental health problems were recorded by GPs, and which treatment (prescriptions for psychotropic medication and referrals to primary and secondary mental health care) was offered to children and adolescents with mental health problems.

GPs' identification and treatment of mental health problems may be different for children and adolescents. Whereas parents play an important role in identifying problems and initiating the use of services for children [17], adolescents' increasing autonomy strengthens their influence on the help-seeking process [19]. Adolescents may be reluctant to seek help for mental health problems [37] and/or to mention their mental health problems to their GP, thereby possibly affecting the chance of being identified as having mental health problems by their GP. Moreover, the treatment of mental health problems of children and adolescents may be different, for instance because of differences in protocols for prescribing psychotropic medication to these age groups. Therefore, results are reported for children (0-12 years) and adolescents (13-18 years) separately.

## Methods

Data about morbidity, prescriptions and referrals were derived from electronic medical records of a representative sample of GPs who participate in the Netherlands Information Network of General Practice (LINH) [38]. Since this network changes in composition yearly, the number of included general practices varies. Table 1 presents the numbers of general practices in LINH from 2004 to 2008 and the numbers of patients (0-18 years) registered within these practices. These numbers refer to patients for whom diagnoses were available. Numbers of patients for whom referral and prescription data were available are reported separately (see Results). In 2006, another Dutch GP registration network was integrated within LINH, which led to an increase in the number of participating GPs. LINH contains a representative sample of Dutch GPs and GP patients in all years.

**Table 1:** Numbers of general practices and registered patients (0-18 years) in 2004-2008

	2004	2005	2006	2007	2008
N general practices	43	42	69	81	82
General practice population^a^	40155	37716	64685	75032	73432
0-12 years	28111	26048	44829	52118	50988
13-18 years	12044	11668	19856	22914	22444
Female	19600	18296	31336	36251	35490
Male	20555	19420	33349	38781	37945

^a ^Numbers of children and adolescents (0-18 years) registered in the included general practices

Diagnoses are recorded using the International Classification for Primary Care (ICPC) [39], which is the standard for coding morbidity in Dutch general practice. ICPC-codes referring to mental health problems (P-codes P01-P99) were selected for this study, as well as the separate diagnoses of anxiety (P01, P74), depression (P03, P76), alcohol abuse (P15, P16) and overactivity (P21).

Prescriptions are recorded using the Anatomical Therapeutic Chemical classification system [40]. Prescriptions for antipsychotics (N05A, including lithium, N05AN01), anxiolytics (N05B), hypnotics and sedatives (N05C), antidepressants (N06A), psychostimulants used for Attention Deficit Hyperactivity Disorder (N06BA04, N06BA09), and alcohol dependence medication (N07BB) were selected.

Referrals to primary (i.e. social workers, primary care psychologists, social psychiatric nurses) and secondary mental health care (i.e. psychiatrists, psychotherapists, ambulatory mental health care organisations, and organisations for addiction treatment) were included in this study. Ambulatory mental health care organisations provide ambulatory care to patients whose mental health problems do not require residential care, but are too complex to be handled in primary care. Care in these organisations is provided by psychotherapists, psychologists and psychiatrists.

GPs in LINH are obliged to record referrals to social workers, primary care psychologists, psychiatrists, and ambulatory mental health care organisations. Recording of referrals to social psychiatric nurses, psychotherapists and organisations for addiction treatment is optional.

LINH does not contain information about psychological management (e.g. counselling or short-term therapy) provided in general practice.

LINH is registered with the Dutch Data Protection Authority. All data are collected and handled according to the data protection guidelines of this authority. According to Dutch legislation, studies using this kind of observational data do not require medical ethical approval.

### Analyses

To investigate the extent to which GPs identify child and adolescent mental health problems, prevalence rates of recorded mental health problems (ICPC-codes within the P-chapter) were determined for 2004-2008. The effects of patient age and gender on recorded prevalence rates, rates of referral and prescriptions in 2008 were analysed using multivariate multilevel logistic regression analyses with two-level hierarchical structured data (patients within general practices), using MLWin 2.02. Time trends in prevalence rates were analysed using multilevel repeated measures logistic models with three-level hierarchically structured data (occasions nested within patients, and patients nested within general practices). Models were adjusted for patient gender and the practice's length of recording. Time trends in prescriptions and referrals were analysed by multilevel logistic regression, using a compound-symmetry model with three-level hierarchically structured data. Models were adjusted for patient gender. Analyses were performed for children (0-12 years) and adolescents (13-18 years) separately.

## Results

### Prevalence of child and adolescent mental health problems recorded in general practice

Rates of children and adolescents who were recorded as having a mental health problem are reported in Table 2. In 2008, 6.6% of children and 7.5% of adolescents were recorded as having a mental health problem (ICPC-code P01-P99). Of the separate diagnoses selected for this study, overactivity was recorded most frequently over the years. Apart from these selected diagnoses, other frequently recorded diagnoses within the P-chapter were enuresis (P12), other concern with behaviour of child (P22), and specific learning problems/delay in development (P24).

**Table 2:** Prevalence of child and adolescent mental health problems recorded in general practice in 2004-2008

	Prevalence per 100					Linear time trend	
	2004	2005	2006	2007	2008	X^2^	p
General practice population^a^	40155	37716	64685	75032	73432		
Mental health problems (P01-P99)							
0-12 years	4.80	4.47	5.45	5.14	6.62	22.06	< 0.001
13-18 years	5.84	4.97	5.94	5.42	7.52	9.15	0.003
Anxiety (P01, P74)							
0-12 years	0.18	0.16	0.24	0.27	0.29	15.94	< 0.001
13-18 years	1.08	0.80	0.94	0.90	1.12	2.90	0.09^b^
Depression (P03, P76)							
0-12 years	0.07	0.04	0.08	0.06	0.09	1.23	0.27
13-18 years	1.07	0.76	0.73	0.59	0.83	4.30	0.04^c^
Alcohol abuse (P15, P16)							
0-12 years	0.00	0.00	0.00	0.00	0.00	0.04	0.84
13-18 years	0.03	0.02	0.04	0.06	0.08	4.34	0.04
Overactivity (P21)							
0-12 years	0.74	0.59	0.70	0.68	0.84	2.00	0.16
13-18 years	0.58	0.56	0.74	0.78	0.98	1.12	0.29

Time trend analyses were adjusted for gender and the practice's length of recording.

^a ^Numbers of youths (0-18 years) registered in the included general practices.

^b ^Although no significant linear trend was found, the prevalence of adolescent anxiety showed a significant quadratic trend (chi-square: 5.47; p = 0.02).

^c ^Although a significant linear trend was found, the prevalence of adolescent depression showed a stronger quadratic trend (chi-square: 8.40; p = 0.004).

The effects of age and gender on the prevalence of recorded mental health problems are reported in Table 3. Girls had an increased chance of being recorded as having anxiety or depression, and a decreased chance of being recorded as having overactivity. Compared to children, adolescents had an increased chance of being recorded as having anxiety or depression. After adjusting for gender, age did not significantly affect the prevalence of recorded overactivity.

**Table 3:** Effects of age and gender on the prevalence of recorded mental health problems in 2008

	Mental health problems (P01-P99)	Anxiety (P01, P74)	Depression (P03, P76)	Overactivity (P21)
Female	0.65 (0.61-0.69)	1.59 (1.29-1.95)	2.35 (1.76-3.12)	0.21 (0.17-0.26)
Adolescent (13-18)	1.18 (1.11-1.26)	3.97 (3.22-4.90)	9.87 (7.01-13.87)	1.18 (1.00-1.40)

Table entries are odds ratios with 95% confidence intervals in parentheses. Male gender and child age (0-12 years old) were used as reference categories. Because of the low prevalence of alcohol abuse (see Table 2), analyses were not performed for this diagnosis.

The prevalence of recorded mental health problems (P01-P99) showed a significant linear increase between 2004 and 2008, for both children and adolescents (Table 2). A similar increase was found for the prevalence of anxiety in children and alcohol abuse in adolescents. The prevalence of recorded anxiety and depression in adolescents showed a significant quadratic trend, whereas no significant trend was found for the prevalence of depression in children and the prevalence of overactivity in both age groups.

### Prescriptions for psychotropic medication

Percentages of children and adolescents who were prescribed psychotropic medication are reported in Tables 4 and 5. In 2008, 15.2% of children and 29.4% of adolescents with mental health problems were prescribed psychotropic medication. In 2008, the majority of children (60.4%) and adolescents (74.7%) who were recorded as having overactivity received a prescription for psychotropic medication. Prescription rates were lower for anxiety and depression (Table 5). Compared to children, adolescents had an increased chance of receiving any prescription for psychotropic medication (see additional file 1).

**Table 4:** Psychotropic prescriptions for youths with mental health problems in 2004-2008

	Prescriptions^a^					Linear time trend	
	2004	2005	2006	2007	2008	X^2^	p
N general practices	49	63	63	74	78		
N patients	2259	2782	3410	4082	4954		
Any psychotropic prescription							
0-12 years	12.97	13.65	12.15	13.32	15.22	1.93	0.16
13-18 years	32.49	31.40	26.38	27.27	29.39	4.16	0.04
Antipsychotics (N05A)							
0-12 years	3.14	2.73	1.92	1.90	2.11	0.93	0.33
13-18 years	4.03	3.39	4.19	3.95	4.05	0.11	0.74
Anxiolytics (N05B)							
0-12 years	1.64	0.70	1.05	0.84	0.89	1.45	0.23
13-18 years	7.68	7.22	5.17	4.69	5.29	6.02	0.01
Hypnotics and sedatives (N05C)							
0-12 years	0.75	1.18	1.14	1.35	1.74	4.22	0.04
13-18 years	4.53	5.36	4.46	4.47	4.76	0.03	0.86
Antidepressants (N06A)							
0-12 years	1.02	1.39	0.44	0.47	0.37	6.80	0.009
13-18 years	11.21	8.10	4.28	4.62	5.65	13.52	< 0.001
Psychostimulants (N06BA04, N06BA09)							
0-12 years	7.99	9.26	8.78	10.33	12.22	8.29	0.004
13-18 years	10.58	12.25	11.94	14.01	15.29	3.07	0.08

Time trend analyses were adjusted for gender. Alcohol dependence medication (N07BB) was not prescribed.

^a ^Percentages of children and adolescents with mental health problems (P01-P99) who received a psychotropic prescription.

**Table 5:** Percentages of youths who received a psychotropic prescription in 2008, specified per mental health problem

	Mental health problems (P01-P99)	Anxiety (P01, P74)	Depression (P03, P76)	Overactivity (P21)
N patients	4954	389	234	743
Any psychotropic prescription				
0-12 years	15.22	11.19	10.87	60.37
13-18 years	29.39	23.58	35.64	74.71
Antipsychotics (N05A)				
0-12 years	2.11	0.00	2.17	6.43
13-18 years	4.05	1.63	3.72	9.58
Anxiolytics (N05B)				
0-12 years	0.89	3.50	0.00	0.21
13-18 years	5.29	14.63	9.57	1.15
Hypnotics and sedatives (N05C)				
0-12 years	1.74	0.00	2.17	4.36
13-18 years	4.76	3.25	5.85	4.21
Antidepressants (N06A)				
0-12 years	0.37	0.00	6.52	1.24
13-18 years	5.65	7.32	25.00	3.07
Psychostimulants (N06BA04, N06BA09)				
0-12 years	12.22	7.69	2.17	56.64
13-18 years	15.29	1.22	2.13	68.97

Prescriptions for alcohol abuse are not reported, because of the low prevalence of alcohol abuse in this sample (see Table 2).

The overall percentage of children with mental health problems receiving a psychotropic prescription did not change significantly over the years, whereas a slight decrease was found for adolescents (Table 4). The percentage of children with mental health problems who were prescribed psychostimulants increased significantly, from 8.0% in 2004 to 12.2% in 2008. When only children with a primary care diagnosis of overactivity were considered, a similar significant increase in prescriptions for psychostimulants was found (chi-square: 5.53; p = 0.02). No significant linear trend in prescriptions of psychostimulants was found for adolescents. Prescriptions for antidepressants decreased over time in both age groups. Prescriptions for hypnotics and sedatives increased over time for children, but not for adolescents, while prescriptions for anxiolytics decreased over time for adolescents, but not for children.

### Referrals to primary and secondary mental health care

Percentages of children and adolescents who were referred to primary or secondary mental health care are reported in Tables 6 and 7. In 2008, 18.9% of children and 22.9% of adolescents with mental health problems were referred to any kind of primary or secondary mental health care (Table 6). Compared to children, adolescents had an increased chance of being referred to primary care (see additional file 1). Over the years, children and adolescents who were referred to other health care providers, were significantly more often referred to secondary than to primary mental health care (p < 0.05).

**Table 6:** Referrals of youths with mental health problems to primary or secondary mental health care in 2004-2008

	Referrals^a^					Linear time trend	
	2004	2005	2006	2007	2008	X^2^	p
N general practices	37	51	37	41	49		
N patients	1818	2416	2278	2136	3157		
**Any referral to mental health care**							
0-12 years	13.83	12.08	14.47	15.50	18.93	10.18	0.001
13-18 years	18.04	20.43	23.83	23.30	22.94	4.21	0.04
**Primary mental health care**							
0-12 years	1.55	1.29	1.90	2.28	3.00	6.98	0.008
13-18 years	3.67	5.42	4.66	4.49	6.07	1.50	0.22
Social work							
0-12 years	0.00	0.00	0.20	0.07	0.24	-	-
13-18 years	1.22	1.26	1.33	0.58	1.04	-	-
Primary care psychologist							
0-12 years	1.55	1.29	1.51	2.21	2.76	5.64	0.02
13-18 years	2.45	3.66	2.53	3.62	4.74	3.35	0.07
Social psychiatric nurse							
0-12 years	0.00	0.00	0.20	0.00	0.00	-	-
13-18 years	0.00	0.50	0.80	0.29	0.38	-	-
**Secondary mental health care**							
0-12 years	12.37	10.84	12.77	13.36	15.98	5.76	0.02
13-18 years	14.98	15.38	19.31	18.96	17.44	1.53	0.22
Psychotherapist							
0-12 years	2.49	1.66	2.23	2.21	4.47	3.81	0.05
13-18 years	2.75	3.15	3.60	3.91	6.07	5.01	0.03
Psychiatrist							
0-12 years	6.87	6.96	7.40	8.24	8.28	1.93	0.16
13-18 years	7.03	7.69	10.39	10.56	7.58	0.43	0.51
Ambulatory mental health care org.							
0-12 years	3.09	2.34	3.27	2.91	3.43	0.05	0.82
13-18 years	5.50	4.54	4.93	4.34	3.70	4.17	0.04
Org. for addiction treatment							
0-12 years	0.00	0.00	0.00	0.00	0.00	-	-
13-18 years	0.00	0.25	0.40	0.43	0.28	-	-

Time trend analyses were adjusted for gender. Since recording of referrals to social psychiatric nurses, psychotherapists, and organisations for addiction treatment is optional, percentages of referrals to these types of care may have been underestimated. Referrals to social work, social psychiatric nurses and organisations for addiction treatment were too low to perform time trend analyses.

^a ^Percentages of children and adolescents with mental health problems (P01-P99) who were referred to primary or secondary mental health care.

**Table 7:** Referrals to primary or secondary mental health care in 2008, specified per mental health problem

	Mental health problems (P01-P99)	Anxiety (P01, P74)	Depression (P03, P76)	Overactivity (P21)
N patients	3157	251	145	500
**Any referral to mental health care**				
0-12 years	18.93	33.98	33.33	20.62
13-18 years	22.94	27.03	43.48	11.43
**Primary mental health care**				
0-12 years	3.00	7.77	13.33	2.46
13-18 years	6.07	9.46	14.78	1.14
Social work				
0-12 years	0.24	0.00	3.33	0.00
13-18 years	1.04	0.68	3.48	0.00
Primary care psychologist				
0-12 years	2.76	7.77	10.00	2.46
13-18 years	4.74	8.11	12.17	1.14
Social psychiatric nurse				
0-12 years	0.00	0.00	0.00	0.00
13-18 years	0.38	0.68	0.00	0.00
**Secondary mental health care**				
0-12 years	15.98	26.21	20.00	18.15
13-18 years	17.44	19.59	31.30	10.29
Psychotherapist				
0-12 years	4.47	7.77	6.67	0.92
13-18 years	6.07	7.43	10.43	1.14
Psychiatrist				
0-12 years	8.28	14.56	10.00	12.00
13-18 years	7.58	8.11	11.30	6.29
Ambulatory mental health care org.				
0-12 years	3.43	3.88	3.33	5.54
13-18 years	3.70	4.73	9.57	2.29
Organisation for addiction treatment				
0-12 years	0.00	0.00	0.00	0.00
13-18 years	0.28	0.00	0.00	0.57

Percentages of children and adolescents who were referred to primary or secondary mental health care providers are reported. Referrals for alcohol abuse are not reported, because of the low prevalence of alcohol abuse in this sample (see Table 2).

Between 2004 and 2008, the percentages of children and adolescents who were referred to any mental health care provider increased significantly (Table 6). In primary care, this increase could mainly be ascribed to an increase in referrals to primary care psychologists for children. Over the years, adolescents were increasingly referred to psychotherapists, and decreasingly to organisations for ambulatory mental health care.

### Combinations of treatment

Psychotropic medication and mental health care referrals were mainly offered separately. Only a minority of children and adolescents (in 2008: 2.1% of children and 4.5% of adolescents) received both types of treatment. A considerable number of youths with mental health problems (in 2008: 66.5% of children and 50.3% of adolescents) received neither psychotropic medication nor were referred to primary or secondary mental health care.

## Discussion

As the majority of Dutch children and adolescents - including those with mental health problems - visit their GP at least once a year [18,20,22], and GPs are usually the first health care providers to be consulted when parents have concerns about their child's health, GPs can play an important role in the identification, treatment and referral of child and adolescent mental health problems. It is unknown, however, to what extent this role is actually fulfilled by GPs. Moreover, several measures to strengthen primary mental health care may have changed the role of GPs in the identification and management of children and adolescents with mental health problems. To investigate the extent to which child and adolescent mental health problems are identified and treated in general practice and whether this has changed over the years, this paper presented a comprehensive overview of mental health care as delivered by Dutch GPs between 2004 and 2008.

### Identification of child and adolescent mental health problems

In 2008, 6.6% of children and 7.5% of adolescents were recorded by their GP as having a mental health problem. This is in sharp contrast with prevalence rates of 14-22% reported previously for mental health problems in the general child and adolescent population [2-7]. A recent study in an adult population (18-65 years) [Verhaak et al., unpublished data] showed a much smaller difference between the prevalence of mental health problems in general practice (12.5%) and the general population (17.5%). In line with previous research [18,21,24,26], these results may indicate that a number of youths with mental health problems are not identified by their GP. Although not all children and adolescents with mental health problems will need additional care, it is important to identify the ones who do.

The difference between the prevalence rates of child mental health problems recorded in general practice and in the general population may partly be explained by the fact that not all youths with mental health problems and/or their parents experience a need for care and/or consult their GP. Parents and youths may not be aware that GPs are an appropriate source of care for mental health problems [25]. Particularly adolescents may be reluctant to seek help for mental health problems [37] and to mention their mental health problems to their GP, for instance because of fear about a lack of confidentiality [25]. This may affect adolescents' chance of being identified as having mental health problems by their GP. However, our data did not confirm this, as the prevalence of recorded mental health problems was not found to be lower for adolescents than for children. This may be an indication of parents' continuing influence on the help-seeking process [19].

When youths or parents do consult their GP, mental health problems are rarely being presented as the main reason for the consultation [41-43]. Even if patients do express their concerns, they generally do this in the context of physical problems, instead of directly relating to the actual problems [22,44]. The limited length of consultations may contribute to this reluctance, as more time is needed to discuss psychological issues [45-47].

Alternatively, GPs may be reluctant to diagnose a child or adolescent with mental health problems because of the stigma associated with the diagnosis. As in many countries, demand for child and adolescent mental health care in the Netherlands exceeds supply, which results in considerable waiting lists [48]. Particularly when the likelihood of obtaining specialist mental health care following referral is limited due to these waiting lists, GPs may not see the benefit of recording youths as having a mental health problem.

The prevalence of child and adolescent mental health problems recorded in general practice increased between 2004 and 2008, which may indicate that the identification of these problems by GPs has improved. It may also indicate that a previously found increase in the prevalence of Dutch children's and adolescents' mental health problems between 1993 and 2003 [3,49] has persisted in later years, or that society's attitude towards mental health problems has changed, decreasing the stigma being attached to these problems and to seeking professional help.

### Treatment of child and adolescent mental health problems

In 2008, 15.2% of children and 29.4% of adolescents with mental health problems were prescribed psychotropic medication. Over the years, psychostimulants were prescribed most frequently. Previous research showed an increase in the use of psychostimulants by children and adolescents during the last two decades [33]. Although these previous results were based on pharmacy dispensing records, which include prescriptions from GPs and psychiatrists, we found a similar increase in the prescriptions for psychostimulants by GPs for children aged 0-12 years. This increase was not reflected by an increase in overactivity recorded in this age group, which may indicate that psychostimulants are increasingly being prescribed to overactive children. Our results further indicate that a previously found decrease in GPs' prescriptions of antidepressants to children and adolescents between 2001 and 2005 [50] persisted in later years. This may be due to the increasing consensus that antidepressant medication should not be used as first-line treatment for children and adolescents with mild to moderate depression, because of its limited - or in some cases even adverse - clinical effects [51,52].

In 2008, 18.9% of children and 22.9% of adolescents with mental health problems were referred to any kind of mental health care. Despite recent reforms in Dutch mental health care, which aimed to make Youth Welfare Work Offices the only entrance point into child mental health care [36], we found an increase in the overall percentage of youths being referred to mental health care by their GP, which indicates that GPs still function as gatekeepers to child mental health care.

Over the years, only a minority of children and adolescents with mental health problems were referred to other health care providers. A considerable number of youths with mental health problems (in 2008: 66.5% of children and 50.3% of adolescents) received neither psychotropic medication nor were referred to primary or secondary mental health care. This may indicate that child and adolescent mental health problems are being undertreated. However, not all youths with mental health problems will need additional care. Watchful waiting may be the appropriate strategy for youths with mild mental health problems [29]. Moreover, these children and adolescents may have been offered some kind of psychological management (e.g. counselling or short-term therapy) in general practice. Data about psychological treatment provided in general practice are lacking, however. Children and adolescents, who *were *referred to other professionals, were mainly referred to secondary instead of primary care. Although this may be appropriate for a minority of youths with severe mental disorders, less severe mental health problems should preferably be treated in primary care [29].

The percentage of children being referred to primary mental health care providers increased over the years. This could be a result of Dutch policy measures to strengthen primary mental health care. However, an increase was also found for referrals of children to secondary mental health care. The latter finding is in line with time trend studies in other European countries, which showed an increase in the use of specialist child mental health care services over the years [53,54].

### Strengths and limitations of the study

Although differences between health care systems require caution to be exercised in generalising our results to other countries, we believe that our study provides valuable insights into the role that GPs can play in the identification and treatment of children and adolescents with mental health problems, which may be useful for other countries in which GPs function as gatekeepers. Many of the issues covered here are also relevant for countries with different health care systems. For instance, in the US, community child health services are provided by paediatricians, who are based within primary care. Children and adolescents with mental health problems have been shown to be frequent attenders in primary care [25,27], which underlines the importance of the primary care setting in the identification and treatment of child and adolescent mental health problems.

Our time trend analyses provide insight into changes in the role of GPs in child mental health care over time, which may be a result of efforts to strengthen primary mental health care. However, to evaluate whether these time trends can actually be ascribed to the Dutch measures to strengthen primary mental health care, a more rigorous study design (e.g. an interrupted time series design with multiple measurements before and after the implementation of the policy measures) would be required. Conducting comparative studies in countries in which similar initiatives have been taken, may provide valuable suggestions about ways to strengthen primary mental health care.

Our results are based on a large dataset, which is representative for the Dutch population and for Dutch general practices regarding the degree of urbanisation and region [38]. GPs in LINH are trained in coding and data are checked for completeness and reliability. GPs traditionally did not record mental health referrals in their clinical work, but are asked to record these referrals as part of LINH. Therefore, data regarding mental health referrals may have been underestimated. Moreover, recording of referrals to social psychiatric nurses, psychotherapists and organisations for addiction treatment is optional, which may have caused these referrals to be underestimated.

Our results regarding the treatment of mental health problems in general practice refer to prescriptions for psychotropic medication and referrals to primary and secondary care. They do not indicate whether any kind of psychological management (e.g. counselling or short-term therapy) has been offered in general practice. With regard to the prescription data, it is unknown whether the GP was the initiator of the prescription or whether the GP repeated a prescription that was initiated by a secondary care health care provider.

We did not evaluate the accuracy of GPs' diagnoses of child and adolescent mental health problems by comparing these diagnoses with a gold-standard diagnostic test. Such comparisons would be useful to investigate whether the prescriptions and referrals matched the severity of the child's problems.

## Conclusions

Although GPs' identification of mental health problems and referrals to primary mental health care have increased, the majority of referrals are still made to secondary care. This may indicate that primary mental health care can be further strengthened.

Firstly, mental health literacy programmes may help to increase young people's and their parents' recognition of mental health problems and raise their awareness that GPs are an appropriate source of care for such problems [55,56].

Secondly, since not all children and adolescents with mental health problems need additional care, GPs have to be able to differentiate between transient problems and problems that will persist or even deteriorate without appropriate care. Additional research is needed to investigate whether youths who are identified by their GP as having a mental health problem, are indeed the ones in need of treatment. Increasing GPs' awareness of the possible occurrence of mental health problems in high risk groups (e.g. frequently attending children, children presenting psychosomatic complaints, or children living in stressful circumstances [24]), may assist them in identifying youths who need additional care. Teaching GPs to use psychosocially oriented communication skills, which facilitate the disclosure of information concerning child mental health, and educating them to actively elicit patients' concerns about mental health problems may also contribute to the identification of these problems [43,47,57].

Efforts to improve the identification of children and adolescents who need additional care are only useful if they are accompanied by adequate options for treatment in primary or secondary care [58,59]. To strengthen the role of primary mental health care, efforts should be focused on developing effective short-term interventions for child and adolescent mental health problems that can be applied in general practice. Although several less intensive interventions for mental health problems (e.g. problem solving treatment, psycho-education, and online interventions) have been found to be feasible in general practice [60], they are mainly focused on adult populations. Additional research about the effectiveness and feasibility of interventions for child and adolescent mental health problems in general practice is therefore needed [23,24,61].

## Competing interests

The authors declare that they have no competing interests.

## Authors' contributions

MZ, CD and RV were involved in formulating the research questions. CD performed the analyses and MZ drafted the manuscript. All authors contributed to the interpretation of the data and critically reviewed the manuscript. All authors read and approved the final manuscript.

## Pre-publication history

The pre-publication history for this paper can be accessed here:

http://www.biomedcentral.com/1471-2296/12/133/prepub

## Supplementary Material

Additional file 1 **Effects of age and gender on psychotropic prescriptions and referrals**. Results of multivariate multilevel logistic regression analyses: the effects of age and gender on psychotropic prescriptions and referrals to primary and secondary mental health care for children and adolescents with mental health problems in 2008. Click here for file
