# Supplementary material for: Child and adolescent mental health care in Dutch general practice: time trend analyses
Source: BMC Fam Pract. 2011 Dec 1;12:133. doi: 10.1186/1471-2296-12-133 (PMC3267656; doi:10.1186/1471-2296-12-133)
Supplement: Additional file 1 — Effects of age and gender on psychotropic prescriptions and referrals. Results of multivariate multilevel logistic regression analyses: the effects of age and gender on psychotropic prescriptions and referrals to primary and secondary mental health care for children and adolescents with mental health problems in 2008. [file 1471-2296-12-133-S1.DOC]

## Additional file 1 – Effects of age and gender on psychotropic prescriptions and referrals

## Table 1 - Effects of age and gender on psychotropic prescriptions for youths with mental health problems in 2008

|  | **Any psychotropic prescription** | **Antipsychotics (N05A)** | **Anxiolytics (N05B)** | **Hypnotics and sedatives (N05C)** | **Antidepressants (N06A)** | **Psychostimulants**  **(N06BA04, N06BA09)** |
| --- | --- | --- | --- | --- | --- | --- |
| Female | 0.58 (0.50-0.68) | 0.42 (0.28-0.63) | 2.24 (1.53-3.29) | 1.47 (1.04-2.07) | 2.42 (1.60-3.66) | 0.29 (0.23-0.36) |
| Adolescent^a^ | 2.55 (2.20-2.96) | 2.20 (1.56-3.11) | 5.56 (3.63-8.53) | 2.63 (1.85-3.74) | 15.01 (8.17-27.60) | 1.51 (1.26-1.81) |

Table entries are odds ratios with 95% confidence intervals in parentheses. Male gender and child age (0-12 years old) were used as reference categories.

^a^ 13-18 years old

## Table 2 - Effects of age and gender on referrals of youths with mental health problems in 2008

|  | **Any referral** | **Primary mental health care** | **Primary care psychologist** | **Secondary mental health care** | **Psychotherapist** | **Psychiatrist** | **Ambulatory mental health care org.** |
| --- | --- | --- | --- | --- | --- | --- | --- |
| Female | 1.38 (1.15-1.66) | 2.35 (1.61-3.44) | 2.48 (1.64-3.73) | 1.19 (0.97-1.45) | 1.79 (1.28-2.50) | 0.88 (0.67-1.17) | 1.15 (0.77-1.72) |
| Adolescent^a^ | 1.21 (1.00-1.46) | 1.98 (1.36-2.89) | 1.61 (1.07-2.42) | 1.06 (0.86-1.30) | 1.33 (0.95-1.88) | 0.85 (0.63-1.13) | 1.09 (0.72-1.64) |

Table entries are odds ratios with 95% confidence intervals in parentheses. Male gender and child age (0-12 years old) were used as reference categories. Because of the low rates of referral to social work, social psychiatric nurses, and organisations for addiction treatment (Table 6), analyses were not performed for these variables.

^a^ 13-18 years old
